# Innovative Multidisciplinary Care Models Provided via Telehealth During the COVID Pandemic

**DOI:** 10.1093/geroni/igab046.1084

**Published:** 2021-12-17

**Authors:** Shahla Baharlou, Lee Lindquist

## Abstract

Integrated and collaborative care lead to better care. Addressing the behavioral and mental health care needs of patients results in better health outcomes. Interdisciplinary and multi-disciplinary approaches to health care delivery yield more effective health care planning. A holistic approach to healthcare sees the individual as more than the sum of diseases. Research studies have supported these assertions and yet, in actual practice, they are often more aspirational than actualized. The COVID-19 pandemic has made it even more difficult to implement collaborative care delivered by varied professional disciplines. This symposium describes efforts to provide more holistic and multidisciplinary care in the primary care geriatrics practice of the Dept. of Geriatrics & Palliative Medicine, Icahn School of Medicine. This New York City practice has 4,500 patients with diverse backgrounds and a median age of 85. In the first paper, Baharlou and her colleagues describe the establishment of an IMPACT collaborative care depression model in the middle of the COVID-19 pandemic. It was adapted to be provided by telephone and uses a different psychosocial intervention than is usually implemented. Hinrichsen and Leipzig outline the successful integration of Cognitive Behavioral Therapy for Insomnia into geriatrics primary care to improve insomnia in older adults and deprescribe sleep medications. Munoz and her colleagues describe the ALIGN program which is an interdisciplinary team effort, informed by the social determinants of health framework, to facilitate access to an array of services delivered virtually because of the pandemic.

